# Petersen’s Hernia Following Laparoscopic Roux-en-Y Gastric Bypass: A Retrospective Case Series of Six Patients

**DOI:** 10.7759/cureus.93799

**Published:** 2025-10-03

**Authors:** Paulo Sousa, Eduarda Magalhães, Jose Pedro Pinto, Joaquim Costa Pereira, Ana Cristina Ribeiro

**Affiliations:** 1 General Surgery, Unidade Local de Saúde de Braga, Braga, PRT; 2 Bariatric Surgery, Unidade Local de Saúde de Braga, Braga, PRT; 3 Colorectal Surgery, Unidade Local de Saúde Braga, Braga, PRT

**Keywords:** acute abdomen, bariatric surgery, internal hernia, laparoscopic gastric bypass, mesenteric defect closure, petersen’s hernia, roux-en-y, swirl sign

## Abstract

Petersen’s hernia is a rare but potentially life-threatening complication following laparoscopic Roux-en-Y gastric bypass (LRYGB). Its clinical presentation is often nonspecific, and radiological findings may be subtle, contributing to diagnostic delays. This retrospective study included all patients diagnosed and surgically treated for Petersen’s hernia at Unidade Local de Saúde de Braga, EPE, between January 2023 and June 2025. A total of six patients (three men, three women) were identified, with a mean age of 44 years (range: 31-64 years). All patients had previously undergone LRYGB. The interval between primary surgery and hernia presentation ranged from 20 days to 12 years. Abdominal pain was a universal symptom, frequently accompanied by nausea or vomiting (67%). Computed tomography (CT) suggested internal hernia in five cases (83%), with the swirl sign being the most commonly observed feature. Surgical exploration was performed via laparoscopy in four cases and laparotomy in two. Petersen’s hernia was confirmed in all patients, with no need for bowel resection. All defects were closed using double-layer, non-absorbable barbed sutures. The mean length of hospital stay was 4.3 days (range: 2-6 days), and no postoperative complications, readmissions, or symptom recurrence were observed during follow-up. Despite advances in imaging, Petersen’s hernia remains a diagnostic challenge that may present years after bariatric surgery. High clinical suspicion and prompt surgical exploration are essential. Routine closure of mesenteric defects and management by bariatric-trained surgeons appear to be critical in minimizing morbidity and improving outcomes.

## Introduction

Bariatric surgery, particularly laparoscopic Roux-en-Y gastric bypass (LRYGB), has become a cornerstone in managing morbid obesity and its associated comorbidities. Despite its efficacy, LRYGB is not without complications, with internal hernias emerging as a significant concern in the postoperative period. Among internal hernias, Petersen’s hernia is notably perilous. It involves the herniation of small bowel loops through the potential space between the mesentery of the alimentary limb and the transverse mesocolon, known as Petersen’s defect. This complication can lead to small bowel obstruction, ischemia, and, if unrecognized, significant morbidity and mortality. The incidence of internal hernias post-LRYGB varies, ranging from 0.2% to 9%, as reported, and Petersen’s hernia constituting a substantial proportion of these cases [1].

The minimally invasive nature of laparoscopic surgery, while beneficial in reducing adhesions, inadvertently increases the risk of internal herniation due to greater bowel mobility. Furthermore, rapid postoperative weight loss can exacerbate mesenteric defects, facilitating herniation [2]. Diagnosing Petersen’s hernia poses a clinical challenge. Patients often present with nonspecific symptoms such as intermittent abdominal pain, nausea, and vomiting. While computed tomography (CT) scans are instrumental in evaluation, their sensitivity is limited, with some studies reporting a sensitivity as low as 28% [3].

Consequently, a high index of suspicion is paramount, and exploratory laparoscopy remains the definitive diagnostic and therapeutic approach. Preventive strategies, notably the closure of mesenteric defects during the initial surgery, have been advocated to mitigate the risk of internal hernias. Evidence suggests that such measures can significantly reduce the incidence of Petersen’s hernia [4].

In this retrospective study, we analyze six consecutive cases of Petersen’s hernia treated at a single institution over a 30-month period, with the objective of characterizing their clinical presentation, diagnostic work-up, intraoperative findings, and surgical outcomes. The aim was also to highlight the variability in presentation and the diagnostic challenges that may delay the recognition of Petersen’s hernia, thereby addressing a gap in the current literature. In addition, we discuss the technical and preventive considerations relevant to reducing the incidence and morbidity of this complication.

## Materials and methods

This retrospective study was conducted at Unidade Local de Saúde de Braga, EPE, and included all patients diagnosed with Petersen’s hernia and treated surgically between January 2023 and June 2025. Cases were identified by a systematic review of the institutional surgical logbook and by searching the institution's electronic medical record using the diagnostic and procedural codes of the International Classification of Diseases, 10th revision (ICD-10) for internal hernia and Petersen’s hernia following LRYGB. Inclusion criteria comprised all consecutive patients with a confirmed intraoperative diagnosis of Petersen’s hernia following laparoscopic Roux-en-Y gastric bypass (LRYGB). No exclusion criteria were applied. Demographic data, clinical presentation, imaging findings, surgical approach, and postoperative outcomes were collected from electronic medical records. Two independent authors performed the data extraction, and discrepancies were resolved by consensus to ensure accuracy and reproducibility.

All patients had previously undergone LRYGB, involving the creation of a small gastric pouch, a biliopancreatic limb, and an alimentary limb in Roux-en-Y configuration, resulting in the formation of two potential internal hernia sites: the mesenteric defect at the jejunojejunostomy and the Petersen’s space between the mesentery of the alimentary limb and the transverse mesocolon. Petersen’s hernia was defined as the herniation of small bowel loops through this latter defect. Operative reports were available for the three patients who underwent their primary LRYGB at our institution, and in all cases, closure of the jejunojejunal mesenteric defect had been performed according to local practice. For the remaining three patients, who were operated abroad (Cases 1, 2, and 4), patients were asked to provide documentation from their original centers. The information obtained confirmed that mesenteric defect closure had not been systematically performed. None of the operative reports reviewed mentioned anatomical variations that could have influenced the risk of herniation.

All CT scans were performed with intravenous contrast, including arterial and portal venous phases, and were evaluated in axial and multiplanar reconstructions. Diagnostic criteria included the presence of mesenteric swirl, vessel crowding, small bowel dilation, and signs of wall hypoenhancement suggestive of ischemia. Surgical exploration was prompted by persistent abdominal symptoms and/or radiologic suspicion of internal herniation. Laparoscopy was the initial approach in most cases, conversion to laparotomy was reserved for situations with hemodynamic instability, unclear anatomy, or lack of technical resources. Intraoperative assessment of bowel viability was based on macroscopic criteria, including color, peristaltic activity, mesenteric pulsations, and Doppler evaluation when required. The decision to close the jejunojejunal mesenteric defect was guided by intraoperative findings and the standard protocol of our institution. In all procedures, the Petersen’s defect was closed using non-absorbable barbed sutures in a running two-layer fashion. Additional closure of the mesenteric defect at the jejunojejunostomy was performed when appropriate.

Postoperative data, including length of hospital stay, complications, and readmissions, were recorded. Follow-up evaluations were conducted through scheduled outpatient visits at one week, one month, three months, and six months after surgery, and subsequently at annual intervals. All patients remain in follow-up until the operating bariatric surgeon decides discharge. Patients were monitored for recurrence of symptoms, new-onset gastrointestinal complaints, or need for further imaging or surgical intervention.

The study received approval from the Institutional Ethics Committee of Unidade Local de Saúde de Braga. Written informed consent for the anonymous use of clinical data and imaging was obtained from all patients.

## Results

A total of six patients were diagnosed with Petersen’s hernia and underwent surgical treatment at our institution between January 2023 and June 2025. All had previously undergone laparoscopic Roux-en-Y gastric bypass (LRYGB). The cohort included four men and two women, with a mean age of 45.5 years (range: 31-64 years) and a mean body mass index (BMI) of 26.9 kg/m² (range: 21.3-34.9 kg/m²). The interval between the index surgery and presentation varied from 20 days to 12 years.

All patients presented with abdominal pain, most frequently localised to the epigastric or lower quadrants. Additional symptoms such as nausea, vomiting, or early satiety were reported in four cases. The onset of symptoms was acute in five patients and subacute in one, who reported symptoms persisting for over eight weeks.

Contrast-enhanced computed tomography (CT) was performed in all cases. Imaging was suggestive of internal herniation in five patients (83%), with the classic “swirl sign” of twisted mesenteric vessels identified in three. Other findings included small bowel dilation, converging mesenteric vessels, and, in one case, global bowel hypoperfusion.

A correlation between clinical presentation and imaging features was noted: patients with acute, severe pain and laboratory abnormalities often demonstrated more pronounced CT changes, such as swirl sign or global hypoperfusion, while those with subacute or intermittent symptoms showed subtle or inconclusive imaging findings.

Five patients were initially approached laparoscopically; one required conversion to laparotomy due to dense adhesions and technical difficulty in visualising the mesenteric root. Another patient underwent emergency primary laparotomy prompted by CT features of diffuse hypoenhancement and signs of ischemia. Intraoperative confirmation of Petersen’s hernia was achieved in all six patients. Notably, two patients exhibited reversible signs of ischemia, and one had no active herniation but venous congestion.

No bowel resections were required. The Petersen’s defect was closed in all patients using a double-layer, non-absorbable barbed suture. In one patient, additional closure of the mesenteric defect at the jejunojejunostomy was performed.

Postoperative outcomes were favourable across the series. The median length of hospital stay was four days (range: two to six days), and no postoperative complications, readmissions, or clinical recurrences were recorded during follow-up, which ranged from one to six months.

The clinical characteristics, radiologic findings, and operative details for each case are presented below and summarised in Table 1.

Case 1

**Figure 1:**
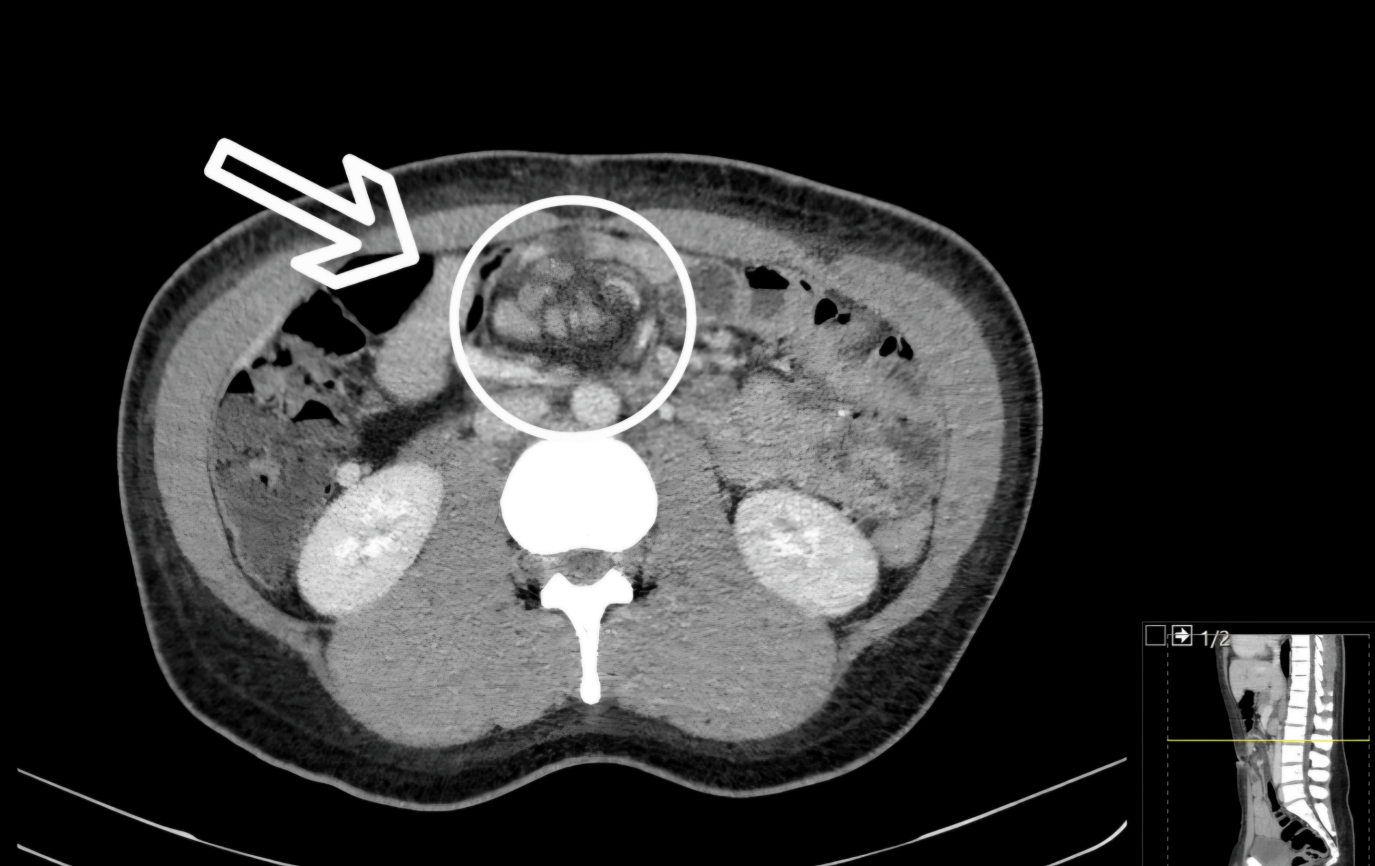
Case 1 - Axial CT scan showing swirl sign suggestive of internal hernia. Contrast-enhanced axial CT image demonstrates the classic “swirl sign” of mesenteric vessels (white circle), consistent with internal herniation. The white arrow highlights the twisted mesenteric fat and vessels, a key radiologic feature in diagnosing Petersen’s hernia.

A 31-year-old male patient, with a history of LRYGB performed 12 years earlier, presented with acute epigastric pain and early satiety. Laboratory evaluation revealed leukocytosis and elevated inflammatory markers. A contrast-enhanced CT scan demonstrated the classic “swirl sign” of mesenteric vessels, highly suggestive of internal herniation through Petersen’s space (Figure 1).

The patient was taken emergently to the operating room. A laparoscopic approach was initially attempted; however, due to dense adhesions and technical difficulty in safely mobilising the bowel, the procedure was converted to an open laparotomy. Intraoperative findings confirmed a Petersen’s hernia with twisted mesentery and distended but viable small bowel loops. The hernia was reduced, and the Petersen’s defect was closed using a two-layer running non-absorbable barbed suture. No bowel resection was necessary. The postoperative course was uneventful, and the patient was discharged on postoperative day 4. He remained asymptomatic at follow-up.

Case 2

A 58-year-old woman, who had undergone LRYGB six years earlier, presented with acute-onset left lower quadrant abdominal pain, associated with nausea and vomiting. Laboratory results revealed leukocytosis (white blood cell count (WBC) 17,000/μL) and elevated C-reactive protein.

**Figure 2:**
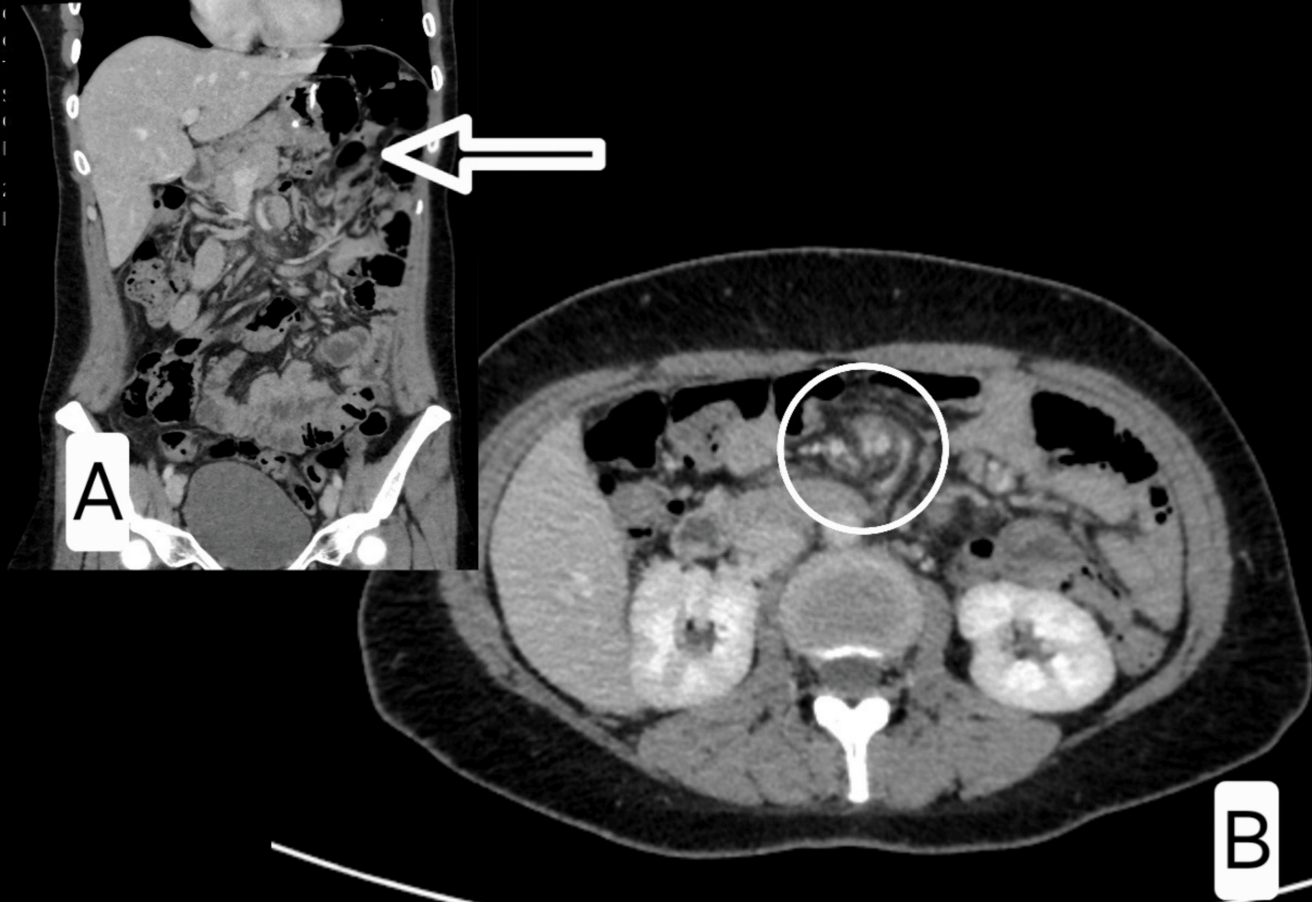
Case 2 - Axial and coronal CT images showing swirl sign and proximal small bowel distension (A) Coronal view showing clustered small bowel loops in the left upper quadrant, posterior to the transverse colon (arrow), suggestive of herniation through Petersen’s space. The white arrow on the coronal image points to the transition zone and the distended proximal small bowel loops, suggestive of mechanical obstruction. (B) Axial view demonstrating the characteristic “swirl sign” (circle), indicative of mesenteric vessel torsion consistent with internal hernia. The white circle highlights the mesenteric swirl sign, indicative of internal herniation. These findings prompted urgent surgical intervention and confirmed Petersen’s hernia with reversible ischemia.

**Figure 3:**
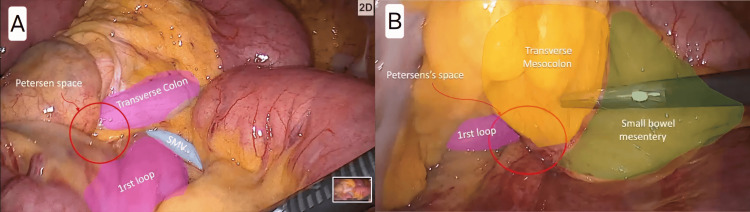
Case 2 - Intraoperative images demonstrating open Petersen’s space without herniation Two laparoscopic images obtained during elective re-exploration: Image A (left): The first jejunal loop, transverse colon, and superior mesenteric vein (SMV) are visualised. The Petersen’s space (red circle) is widely open, without visible herniated bowel. Image B (right): A broader anatomical view showing the relationship between the transverse mesocolon, small bowel mesentery, and the first loop, outlining the Petersen’s defect in its classic configuration. These findings supported the decision for prophylactic closure of the Petersen’s space in the absence of active herniation.

Contrast-enhanced abdominal CT showed swirling of mesenteric vessels and dilated small bowel loops, highly suggestive of internal herniation through Petersen’s space (Figures 2A, 2B). The patient underwent emergency laparoscopy, which confirmed a Petersen’s hernia with venous congestion and distension of small bowel loops. The herniated segments were viable, and perfusion normalised after reduction (Figure 3).

The Petersen’s defect was closed using a running non-absorbable barbed suture in two layers. No bowel resection was needed. Postoperative recovery was uncomplicated, with discharge on day 4. The patient remained well at follow-up.

Case 3

A 48-year-old woman presented to the emergency department with colicky epigastric pain radiating to the back, accompanied by nausea and vomiting. She had undergone LRYGB four years prior. Physical examination showed upper abdominal tenderness without peritoneal signs. Laboratory analysis revealed mild leukocytosis.

**Figure 4:**
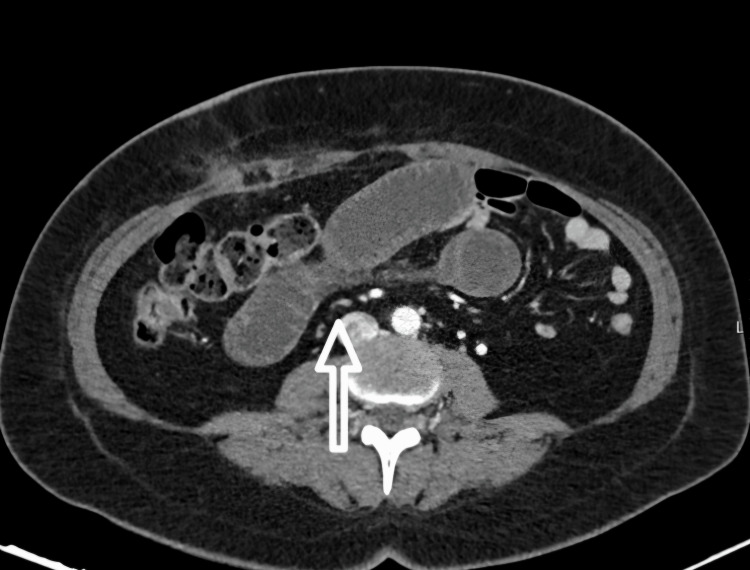
Case 3 - Axial CT scan showing dilated small bowel loop with transition zone Contrast-enhanced axial CT image revealing a markedly distended proximal small bowel loop (highlighted by the white arrow), with a subtle transition zone posterior to the superior mesenteric vessels. These findings were suggestive of mechanical obstruction and prompted urgent laparoscopic exploration, which confirmed Petersen’s hernia with early reversible ischemia.

**Figure 5:**
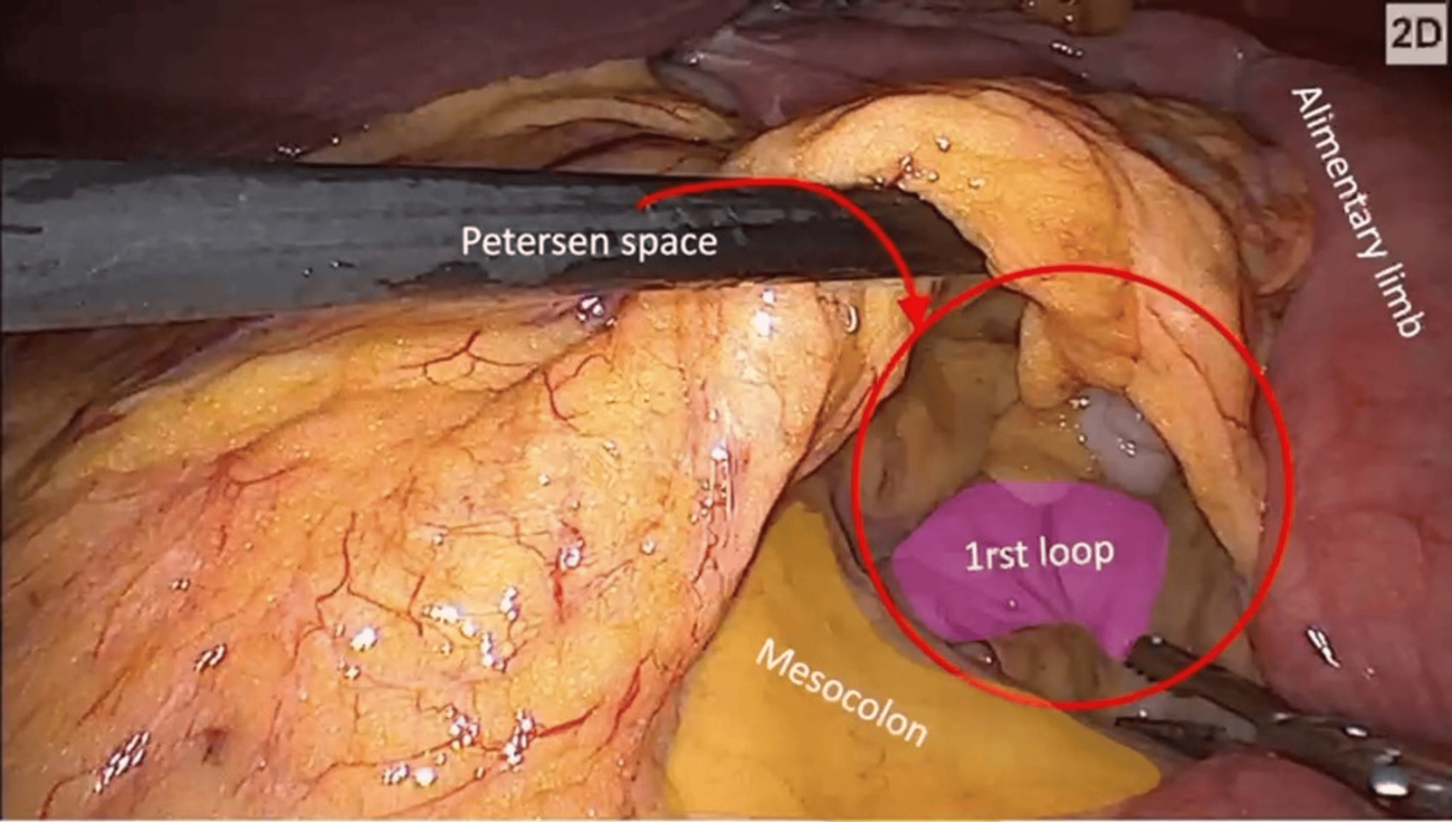
Case 3 - Intraoperative image showing Petersen’s hernia with partially herniated small bowel loop Laparoscopic view illustrating Petersen’s space, bordered superiorly by the transverse mesocolon and inferiorly by a herniated jejunal loop (labeled “1st loop”). The alimentary limb is seen coursing anteriorly. This image highlights the anatomical window responsible for the internal herniation, which led to reversible ischemia resolved after reduction and closure of the defect.

Contrast-enhanced CT revealed dilated jejunal loops and a transition point in the left upper quadrant (Figure 4), suggestive of Petersen’s hernia. Urgent laparoscopy confirmed herniation of small bowel loops through Petersen’s space, with mesenteric venous congestion but no ischemia (Figure 5).

The hernia was reduced, and the Petersen’s defect was closed with a running non-absorbable barbed suture in two layers. No additional defects were identified, and no bowel resection was required. The patient had an uneventful recovery and was discharged on postoperative day 3. She remained asymptomatic at six-month follow-up.

Case 4

A 38-year-old woman, with a history of LRYGB performed abroad, presented with a three-week history of intermittent right upper quadrant pain. Clinical evaluation revealed no signs of peritonitis. Laboratory analysis was unremarkable.

**Figure 6:**
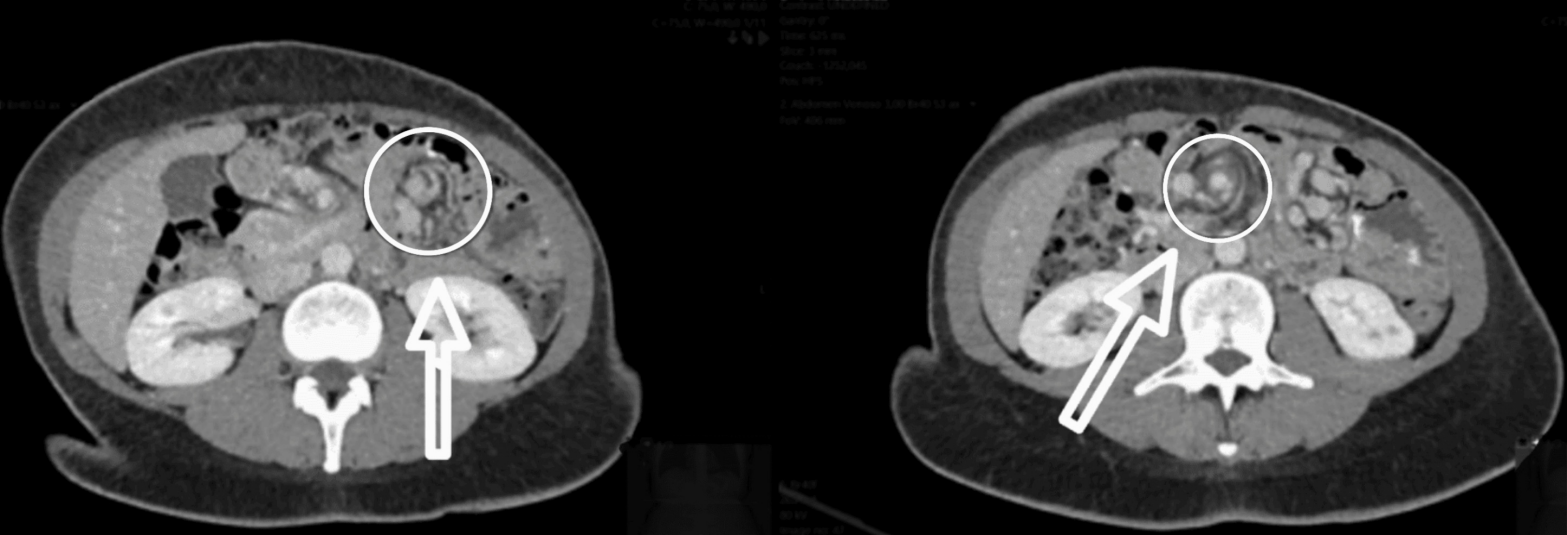
Case 4 - Axial CT scans showing progressive mesenteric swirling suggestive of Petersen’s hernia Two consecutive axial slices of a contrast-enhanced abdominal CT. The white circle delineates the twisted mesenteric vessels and fat (swirl sign), consistent with internal hernia. The white arrows highlight the progressive rotation of the mesentery from one slice to the next (Image A to Image B). These radiologic features support the diagnosis of Petersen’s hernia, even in the absence of overt bowel obstruction.

**Figure 7:**
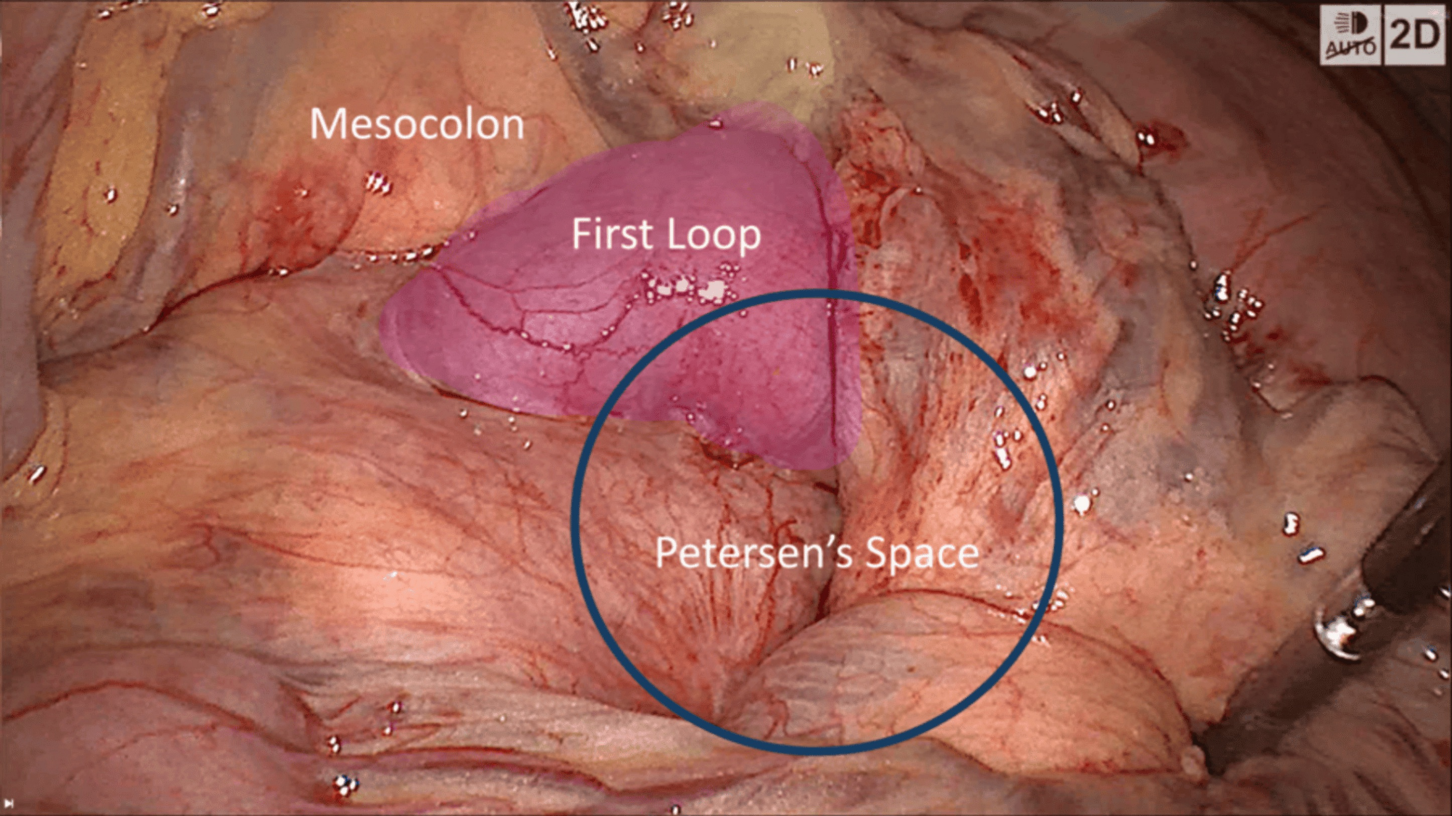
Case 4 - Intraoperative view showing a widely open Petersen’s space Laparoscopic image demonstrating an open Petersen’s space (blue circle), bordered by the transverse mesocolon (labelled “Mesocolon”) and the first jejunal loop (labelled “First Loop”). The hernia defect appears wide and unobstructed, with no bowel content herniated at the time of inspection. This image corresponds to the prophylactic closure performed in the absence of active herniation.

Contrast-enhanced abdominal CT demonstrated mesenteric vessel crowding and a swirling pattern, suggestive of internal herniation through Petersen’s space (Figure 6). Urgent laparoscopy revealed distended but viable small bowel loops and a widely open Petersen’s defect, without evidence of strangulation or ischemia (Figure 7).

The defect was closed in two layers using a non-absorbable barbed suture. The patient had an uneventful postoperative course and was discharged on day 6. At the six-month follow-up, she remained asymptomatic, with no signs of recurrence.

Case 5

A 50-year-old man, 12 months after undergoing LRYGB, presented with six days of colicky epigastric pain without associated vomiting. Laboratory tests showed no leukocytosis or elevated inflammatory markers.

**Figure 8:**
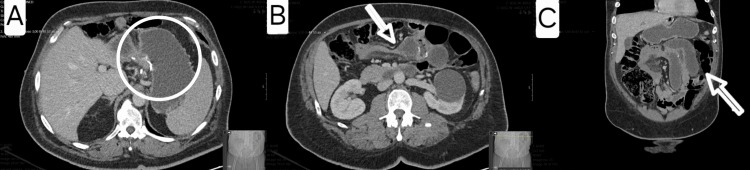
Case 5 - Triptych of abdominal CT images showing inconclusive but suggestive findings of internal hernia Sequential CT images from a contrast-enhanced abdominal scan: Image A (axial): The white circle highlights a congested mesenteric root with slight crowding of vessels. Image B (axial): The white arrow points to distended proximal small bowel loops. Image C (coronal): The white arrow indicates abrupt transition of caliber and clustered loops in the left upper quadrant. Despite the absence of a definitive swirl sign, these findings raised clinical suspicion for internal herniation. Surgical exploration confirmed mesenteric venous congestion without frank herniation, and Petersen’s space was closed prophylactically.

Contrast-enhanced abdominal CT revealed mildly dilated small bowel loops and subtle signs of mesenteric congestion, but no definitive swirl sign (Figure 8). Due to persistent symptoms and a high index of suspicion, laparoscopic exploration was performed.

No herniated bowel was identified intraoperatively, but mesenteric venous congestion was evident in the region of Petersen’s space. In the absence of active herniation, the defect was closed prophylactically using a running suture with non-absorbable barbed material. The patient was discharged on postoperative day 3 without complications. At the three-month follow-up, he remained asymptomatic.

Case 6

A 64-year-old man, with a history of LRYGB performed 12 months earlier, presented with sudden and severe lower abdominal pain. Laboratory findings revealed marked leukocytosis (22,000/μL) and elevated inflammatory markers.

**Figure 9:**
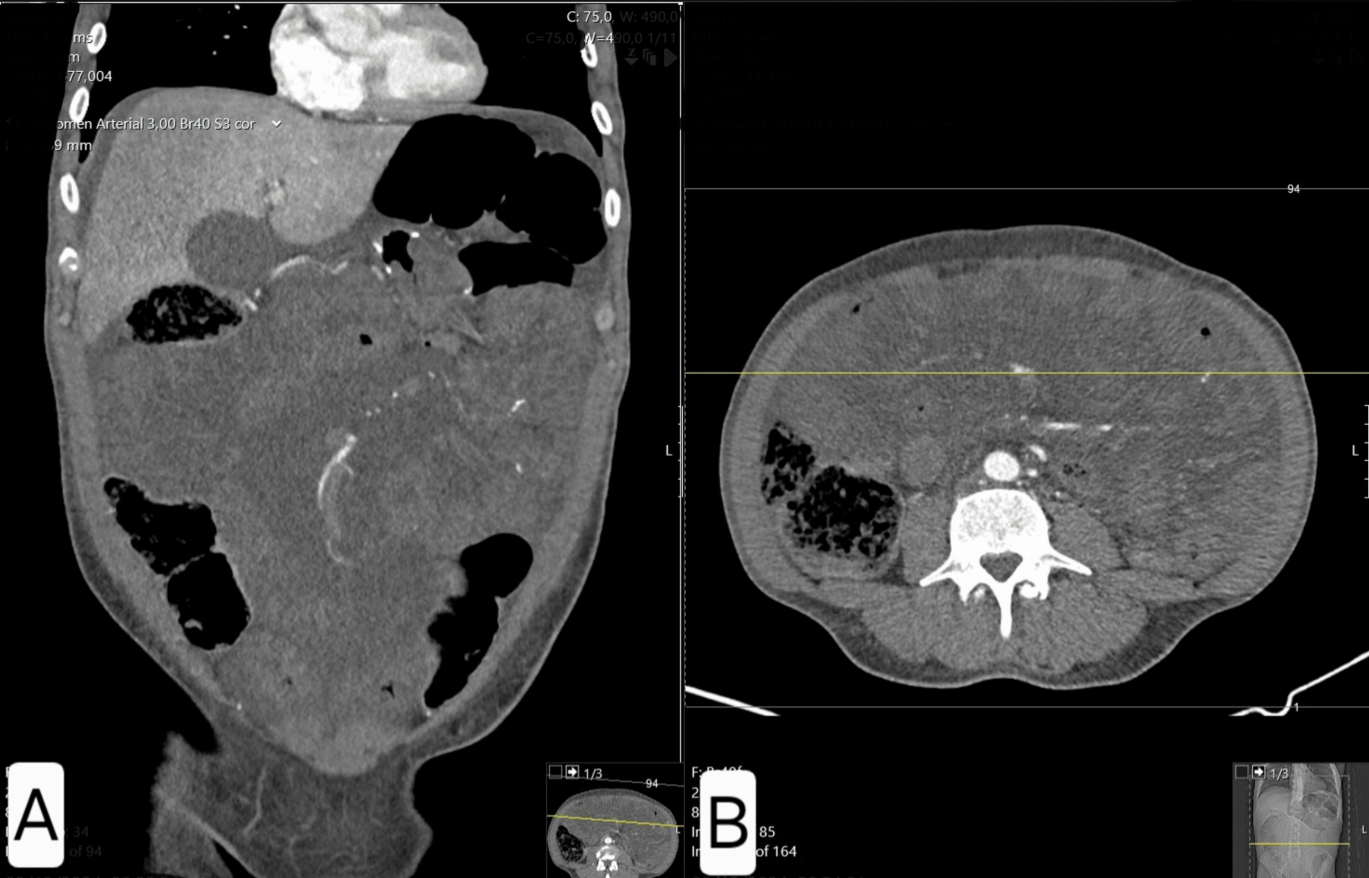
Case 6 - CT findings suggesting extensive small bowel hypoperfusion Image A (coronal view): Distended small bowel loops with global hypoenhancement and mural thickening. Image B (axial view): Reduced contrast enhancement throughout the mid-abdominal small bowel, raising suspicion of diffuse ischemia. These findings were corroborated intraoperatively and prompted urgent surgical intervention.

Contrast-enhanced CT imaging demonstrated globally reduced enhancement of small bowel loops, suggestive of diffuse mesenteric hypoperfusion and raising concern for acute ischemia (Figure 9). Due to the severity of symptoms and imaging findings, the patient underwent emergency laparotomy.

Intraoperatively, a Petersen’s hernia was identified with small bowel loops showing significant venous congestion but no irreversible ischemia. The hernia was reduced, and bowel perfusion normalised promptly. The Petersen’s defect was closed in two layers using non-absorbable barbed suture (V-Loc™), with additional reinforcement of the mesenteric defect. The patient had an uneventful recovery and was discharged on postoperative day 5. At one-month follow-up, he remained asymptomatic.

As detailed in Table 1, this retrospective case series includes six patients diagnosed with Petersen’s hernia following laparoscopic Roux-en-Y gastric bypass (LRYGB). The mean age was 45.7 years (range: 31-64 years), and the majority were male (four out of six, 67%). The mean body mass index (BMI) at the time of diagnosis was 26.9 kg/m² (range: 21.3-34.9 kg/m²).

**Table 1:** Clinical, Radiological, and Surgical Characteristics of Six Patients Diagnosed with Petersen’s Hernia Following Laparoscopic Roux-en-Y Gastric Bypass This table summarises the demographic features, symptom duration, radiologic findings, surgical approach, intraoperative findings, and treatment details of six patients diagnosed with Petersen’s hernia after laparoscopic Roux-en-Y gastric bypass. Cases are presented in chronological order. Computed tomography (CT) findings included the swirl sign in three patients and signs of hypoperfusion or obstruction in the remaining. Two patients required conversion to or primary laparotomy. No bowel resections were necessary. All mesenteric defects were closed intraoperatively.

Case	Sex	Age (years)	BMI (kg/m²)	Time Since RYGB	Main Symptoms	CT Findings	Duration of Symptoms (days)	Closure of mesenteric defect at index surgery	Surgical Approach	Intraoperative Findings	Treatment	Hospital Stay (days)	Follow-Up	Follow-up duration
1	Male	31	25.1	12 years	Epigastric pain, early satiety	Swirl sign	1	No	Converted to laparotomy	Twisted mesentery, no ischemia	Hernia reduction, defect closure	4	No complications reported	6 months
2	Female	36	25.8	10 years	Postprandial pain, nausea	Swirl sign	62	No	Laparoscopy	Open Petersen’s defect, no herniation	Prophylactic defect closure	2	No complications reported	1 year
3	Female	54	34.9	20 days	Acute epigastric pain, vomiting	Small bowel dilation	1	Yes	Laparoscopy	Herniated bowel with reversible ischemia	Hernia reduction, defect closure	5	No complications reported	2 years
4	Female	38	21.3	5 years	RUQ colicky pain	Converging mesenteric vessels	21	No	Laparoscopy	Distended loops, open defect	Hernia reduction, defect closure	6	No complications reported	1 year
5	Male	50	26.3	27 months	Colicky epigastric pain	Inconclusive	6	Yes	Laparoscopy	No herniation, mesenteric congestion	Prophylactic defect closure	3	No complications reported	6 months
6	Male	64	28.0	12 months	Sudden intense lower abdominal pain, leukocytosis	Global hypoperfusion; suspected ischemia	<1	Yes	Laparotomy	Petersen’s hernia with transient ischemia, no necrosis	Hernia reduction; two-layer V-Loc™ closure of Petersen’s and mesenteric defects	5	No complications reported	3 months

The interval between the primary bariatric procedure and hernia presentation ranged from 20 days to 12 years, underscoring the highly variable latency of this complication. The median symptom duration prior to surgical intervention was 3.5 days (range: less than 1 to 62). Three patients presented acutely within 24 hours, whereas one had a protracted clinical course of two months.

All patients reported abdominal pain, most commonly epigastric or colicky in nature. Nausea and vomiting were present in four cases (67%). The laboratory findings were variable in the six cases and are summarized in Table 2. Contrast-enhanced computed tomography (CT) was performed in all patients and was suggestive of internal hernia in five (83%). The most frequently reported radiological features were the swirl sign, bowel dilation, or global hypoperfusion.

**Table 2:** Summary of Laboratory Findings at Time of Diagnosis Baseline laboratory parameters for the six patients diagnosed with Petersen’s hernia following laparoscopic Roux-en-Y gastric bypass. Mild leukocytosis and elevated neutrophil count were observed in most cases. No patient presented with severe renal dysfunction or major electrolyte imbalance. C-reactive protein levels were mildly elevated in all but one case. Values refer to the most recent blood tests obtained prior to surgical intervention. Normal reference ranges are provided for comparison.

Parameter	Reference Range	Case 1	Case 2	Case 3	Case 4	Case 5	Case 6
Hemoglobin (Hb, g/dL)	11.9-15.6 (F)/13.5–17.0 (M)	11.9	11.3	14.6	15.6	14.9	15.5
Leukocytes (10³/µL)	4.0-11.0	5.2	6.4	14.2	7	6.5	12.2
Neutrophils (%)	40-75%	60	47.2	87.4	73.2	72.7	88.7
Platelets (10³/µL)	150-400	270	238	263	262	238	241
CRP (mg/L)	<5.0	1.49	0.7	1.69	1.66	2.85	2.85
Creatinine (mg/dL)	0.70-1.20	0.7	0.7	0.9	0.8	0.8	0.7
Urea (mg/dL)	19-49	26	22	37	40	50	54
Sodium (mmol/L)	136-145	138	138	139	142	144	137
Potassium (mmol/L)	3.5-5.1	4.5	4.9	5	4.9*	4	4.7
Chloride (mmol/L)	98-107	107	108	108	106	102	103

Five patients were initially approached laparoscopically, with one requiring intraoperative conversion to laparotomy due to technical difficulty. One patient underwent primary laparotomy based on clinical or radiological suspicion of bowel ischemia. Petersen’s hernia was confirmed in four cases. In the remaining two, intraoperative findings included mesenteric congestion without herniation and an open Petersen’s defect without active herniation, respectively.

No bowel resections were performed. The Petersen’s defect was closed in all cases using a non-absorbable barbed suture, with additional closure of the mesenteric defect at the jejunojejunostomy in one patient. In four patients, the hernia was reduced prior to closure, while in two cases the closure was performed prophylactically.

The median hospital stay was four days (range: two to six days). No postoperative complications, readmissions, or clinical recurrences were recorded during the follow-up period. The follow-up duration for each patient ranged from three months to two years, as detailed in Table 1. All patients remain under surveillance and continue to be followed until discharge is determined by the treating surgeon.

## Discussion

Petersen’s hernia remains one of the most elusive and potentially hazardous complications following LRYGB. In our case series, we observed a wide range of clinical and temporal variability, with the interval between the primary bariatric procedure and hernia presentation ranging from 20 days to 12 years. This highlights the long-term and unpredictable nature of this complication. Previous studies have similarly reported a broad temporal spectrum. Geubbels et al. described internal hernias presenting anywhere from the immediate postoperative period to as late as 32 months, with a mean of nine months between surgery and diagnosis [5]. In other series such as those by Carmody et al. [6] and Al-Mansour et al. [7], cases of Petersen’s hernia were documented up to four and seven years after the initial surgery, respectively.

In our patients, epigastric or upper abdominal pain was a universal symptom, frequently associated with nausea or vomiting (present in 67%). This presentation is consistent with previously published cohorts, in which abdominal pain is the predominant clinical finding [5,8]. Notably, the duration of symptoms prior to diagnosis was highly variable, ranging from one to 62 days, with a median of 3.5 days. Three patients presented acutely within 24 hours, while others had more protracted or intermittent complaints. This variability is comparable to findings reported in other series, notably by Al-Mansour et al. [7], where the duration of symptoms also ranged widely and often contributed to diagnostic delay.

Computed tomography (CT) remains the cornerstone imaging modality in the evaluation of internal hernias following LRYGB, particularly given the often vague and intermittent clinical presentations [9]. In our series, CT imaging revealed suggestive findings in five out of six patients (83%), with the “swirl sign” being the most observed feature. This radiologic finding - characterised by rotation of the mesenteric vessels and fat - has been consistently associated with internal hernias in the literature and was identified in three of our six cases. Although the swirl sign is not pathognomonic, it is considered one of the most reliable indicators of mesenteric volvulus or internal herniation. Lockhart et al. described the swirl sign as the strongest single predictor of internal hernia among a panel of seven CT features, reporting a sensitivity up to 83% and specificity up to 89% when interpreted by experienced radiologists [10].

However, despite these promising figures, CT may still yield inconclusive or subtle findings, particularly in cases of intermittent or self-reducing hernias. In our cohort, one patient presented with persistent symptoms but inconclusive CT findings; intraoperatively, mesenteric congestion without frank herniation was identified. Another patient (Case 6) exhibited a rare and atypical pattern of global small bowel hypoperfusion, raising concern for widespread ischemia. This underscores the importance of integrating clinical suspicion with imaging and proceeding to diagnostic laparoscopy when uncertainty persists, as imaging alone may not reliably exclude internal herniation [11]. In our cohort, acute presentations were more likely to correlate with striking radiologic features such as mesenteric swirl or global hypoperfusion, whereas protracted or intermittent symptoms were associated with subtler or inconclusive CT findings. This reinforces the need to integrate clinical suspicion with imaging rather than relying on radiology alone.

Comparison between early (≤12 months) and late (more than five years) presentations revealed some differences. Early cases (Cases 3, 5, and 6) typically presented with acute abdominal pain, laboratory abnormalities, and in one case global hypoperfusion requiring primary laparotomy. In contrast, late cases (Cases 1, 2, and 4) often manifested with intermittent or subacute symptoms, and CT abnormalities were less marked. Although outcomes were favorable in both groups, early presentations were more likely to require laparotomy, whereas late cases were usually managed successfully by laparoscopy.

In our case series, two patients required laparotomy. One case (Case 1) was converted due to intra-abdominal adhesions. The other (Case 6) was managed as an emergency by a general surgery team and underwent primary laparotomy due to suspected ischemia. While the latter case was not due to technical challenges such as adhesions, it likely reflected the absence of standardised protocols and the inherent difficulty in managing complex post-bariatric anatomy without specialised training. This observation aligns with findings from Parakh et al., who reported high conversion rates during the initial adoption of laparoscopic techniques, later reduced following the implementation of structured approaches [12]. Similarly, Kollmann et al. demonstrated a significant decrease in conversion rates - from 52.6% to 5.6% - after introducing institutional standards and ensuring that bariatric-trained surgeons were involved in the management of these patients [13]. These data collectively emphasise that consistent protocols and multidisciplinary collaboration play a central role in optimising outcomes and minimising the need for open conversion, especially in the acute setting.

No patients in our series required bowel resection, although one had early signs of ischemia that resolved after reduction. Mesenteric defect closure was performed in all patients. In four cases, closure followed hernia reduction, while in two patients, the closure was prophylactic in the absence of active herniation. This practice is supported by multiple studies and guidelines [14,15]. Stenberg et al. demonstrated in a randomised controlled trial that systematic closure significantly reduces the risk of internal herniation [14]. Additional evidence from the International Federation for the Surgery of Obesity and Metabolic Disorders (IFSO) Global Registry reinforces this recommendation as part of standard surgical technique [16].

Postoperative outcomes were favourable in all cases. The median length of stay was four days (range: two to six days), with no reported complications or readmissions. These findings underscore the benefit of early surgical intervention in preventing morbidity. Our data reinforce key principles in managing Petersen’s hernia: maintain high clinical suspicion in patients with a history of LRYGB who present with abdominal pain, regardless of how remote the surgery was, and interpret CT scans with caution and proceed to diagnostic laparoscopy without delay when clinical suspicion persists. Moreover, the routine closure of mesenteric defects should be considered a standard and preventive measure to minimise the occurrence of this potentially life-threatening complication.

This study has several limitations. It is a retrospective single-center case series with a small number of patients and relatively short follow-up. Operative reports from patients operated abroad were retrieved through patient request but may not provide the same level of detail as institutional records. In addition, reproducibility of the diagnostic process may be limited by the absence of a standardised CT imaging protocol and interpretation criteria across radiologists, which could influence diagnostic accuracy and interobserver agreement. These constraints limit the generalizability of our findings, although they remain consistent with previously published data.

## Conclusions

Petersen’s hernia remains an uncommon yet potentially life-threatening complication after laparoscopic Roux-en-Y gastric bypass, with a highly variable timeframe and often non-specific clinical presentation. This case series underscores the diagnostic complexity, particularly in patients with vague or protracted abdominal symptoms. While contrast-enhanced CT is invaluable in the initial evaluation, its limitations necessitate a low threshold for diagnostic laparoscopy when clinical suspicion persists.

Systematic closure of mesenteric defects continues to be the cornerstone of prevention and should be considered standard practice. Additionally, operative management by bariatric-trained surgeons, within the context of structured institutional protocols, appears to reduce intraoperative complexity and the need for conversion, especially in emergent scenarios. Sustained clinical vigilance, prompt surgical intervention, and adherence to preventive strategies remain essential to reducing the morbidity associated with this serious postoperative complication.

Nevertheless, the interpretation of our results is constrained by the small sample size, retrospective single-center design, and relatively short follow-up. Our findings should therefore be interpreted with caution and regarded as hypothesis-generating, reinforcing the need for larger prospective multicenter studies. Larger prospective multicenter studies are warranted to better define the true incidence, risk factors, and long-term outcomes of Petersen’s hernia.
